# Online food delivery systems and their potential to improve public health nutrition: a response to ‘*A narrative review of online food delivery in Australia’*

**DOI:** 10.1017/S1368980021000926

**Published:** 2021-03-12

**Authors:** Tessa Delaney, Luke Wolfenden, Rebecca Wyse

**Affiliations:** 1 School of Medicine and Public Health, University of Newcastle, Callaghan, NSW 2308, Australia; 2 Hunter New England Population Health, Wallsend, NSW 2287, Australia Email tessa.delaney@health.nsw.gov.au; 3 Hunter Medical Research Institute, New Lambton Heights, NSW 2305, Australia; 4 Priority Research Centre for Heath Behavior, University of Newcastle, Callaghan, NSW 2308, Australia

Worldwide, there are more than 1·2 billion users of online food delivery (OFD) systems (e.g. UberEats, Menu log)^(1)^. As such, we read with great interest the review by Bates *et al.* ^(2)^ in the June 2020 edition of Public Health Nutrition that demonstrated the rapid increase in use of such systems within Australia and highlighted their importance in accessing food in the future (e.g. projected growth of $AU570 million by 2024). The review described the impact, both positive and negative, that these systems can have on public health nutrition and discussed the potential of embedding choice architecture interventions within these systems, as a way to encourage the purchase of healthier menu items at a population level.

One specific OFD system that we believe represents a particularly potent infrastructure to improve public health nutrition in Australia is online school canteen ordering systems. These systems enable students and parents to access the school canteen menu online, and select and pay for meals, drinks and snacks for consumption during school breaks. Online canteens offer a number of unique advantages over other OFD systems as a tool for health improvement. First, the integration of choice architecture interventions in online canteen ordering systems provides access to large and increasing proportions of the child population. For example, canteens are the largest food provider for children^(3)^, and online canteens are becoming increasingly common with two providers alone servicing 2100 schools across Australia^(4,5)^, equivalent to ∼20 % of Australian schools^(6)^.

Second, school food services are more amenable to government regulation or mandatory policy to regulate the availability and nutritional quality of foods, and the use of specific strategies to promote healthy food consumption^(7)^. Policies that currently exist in all Australian states and territories include strategies that require the prominent placement or promotion of healthy foods at the physical point of purchase within school canteens^(8)^. The extension of such policies and regulations to virtual environments would seem warranted and provide policy concordance.

Third, the integration of choice architecture strategies such as labelling, product placement and prompting would be an effective strategy in modifying dietary habits during critical periods in child development. We have found such strategies to be acceptable to school principals (>70 %)^(9)^ and effective at increasing student purchases of healthier foods from online canteens^(10)^. For example, a cluster randomised controlled trial involving 2714 primary school students found significant reductions in the average energy (–572 kJ; *P* < 0·001), saturated fat (–2·38 g; *P* < 0·001) and Na (–231 mg; *P* = 0·005) content of students’ lunch orders following a 2-month choice architecture intervention without any loss of canteen revenue^(10)^.

Finally, although online canteens represent an appealing OFD system whereby choice architecture interventions could be integrated with relative ease, we have identified a number of factors that may limit their implementation at scale. For example, online product databases that classify and label foods according to school food policies provide promising infrastructure that would enable OFD systems to objectively label menu items. However, anecdotally, the ability of OFD systems to freely access government-owned databases is limited, due to licencing agreements on who can access data. Making such databases widely available, and supporting their adoption with government policy, would provide the required technology to enable rapid translation of choice architecture interventions by OFD systems. Furthermore, whilst mandatory menu labelling requirements exist in the broader fast food sector^(11)^, no such requirements are included in Australian school food policies. The application of menu labelling has been identified by parents as a strategy that would influence canteen purchases for their children^(12)^ and would likely facilitate uptake of choice architecture interventions by schools and OFD systems.

In conclusion, the review by Bates *et al.* provides an important contextual overview of OFD system use in Australia and is particularly timely given the disruption of access to physical food environments following recent COVID-19 lockdowns and restrictions. OFD systems present an unprecedented opportunity to reach millions of consumers at the point of purchase and are important for future public health nutrition. As such, further research is needed to better understand and address barriers and facilitators to using OFD systems to deliver public health nutrition interventions to improve food selection behaviours of the population.
